# Effects of Delta-Aminolevulinic Acid Dehydratase Polymorphisms on Susceptibility to Lead in Han Subjects from Southwestern China

**DOI:** 10.3390/ijerph9072326

**Published:** 2012-07-02

**Authors:** Yuelin Yang, Jin Wu, Pin Sun

**Affiliations:** 1 Department of Occupational Hygiene, West China Fourth Hospital of West China Public Health Institute, Sichuan University, 18 Renmin Nanlu, Chengdu 610041, China; 2 Department of Internal Medicine, West China Fourth Hospital of West China Public Health Institute, Sichuan University, 18 Renmin Nanlu, Chengdu 610041, China; Email: jinwuSCU@yahoo.cn; 3 Department of Occupational Health, School of Public Health, Fudan University, 130 Dongan Road, Shanghai 200032, China; Email: sunpinFD@yahoo.com.cn

**Keywords:** lead poisoning, occupational exposure, polymorphism, delta-aminolevulinic acid dehydratase, Chinese

## Abstract

This study is to determine the distribution of the delta-aminolevulinic acid dehydratase (ALAD) polymorphism among Han subjects of the Chinese population and to study whether the polymorphism in the ALAD gene modifies the toxicity of lead in lead-exposed workers. For this purpose we conducted a cross-sectional study on 156 Chinese workers who were exposed to lead in lead-acid battery and electric-flex manufacturing plants. The authors found that the allele frequencies of ALAD1 and ALAD2 were 0.9679 and 0.0321, respectively. Workers with the ALAD 1-1 genotype were associated with higher blood lead levels than those with the ALAD 1-2 genotype. Blood and urine lead levels were much higher in storage battery workers than in cable workers. The self-conscious symptom survey showed that the incidences of debilitation, amnesia and dreaminess were much higher in those had more than five years of tenure or contact with lead on the job within the ALAD 1-1 genotype subgroup. Laboratory examinations showed that serum iron and zinc levels in workers’ with the ALAD 1-2 genotype were higher than those with the ALAD 1-1 genotype, especially in storage-battery workers. Correlation analysis indicated that the blood lead level negatively correlated with serum calcium, iron and zinc level. The data of this study suggest that the ALAD gene polymorphism and serum ion levels may modify the kinetics of lead in blood. Therefore, the authors recommend that an adequate intake of dietary calcium, iron, and zinc or the calcium, iron, and zinc supplementation should be prescribed to Chinese lead exposed workers.

## 1. Introduction

Lead is a known toxicant and remains a persistent environmental health threat [1]. Occupational exposure to lead is most often encountered at lead smelters and battery manufacturing facilities. Exposure to lead can result in significant adverse health effects to multiple organ systems. One of lead's primary effects is hematotoxicity, specifically inhibition of heme synthesis. Lead inhibits three enzymes in the heme biosynthesis pathway: delta-aminolevulinic acid dehydratase (ALAD), coporphyrinogen oxidase, and ferrochelatase, but its effects on ALAD are most profound [2]. ALAD catalyses the second step of heme synthesis and is encoded by a gene located on chromosome 9q34 [3].

However, exposure markers such as blood/urine lead levels or even bone lead levels could not always explain workers’ health status, because individual susceptibilities to lead toxicity may play an important role in lead toxicology and physiology. Gene polymorphism is an important factor that affects human susceptibility to toxins [4,5]. The human ALAD is a polymorphic enzyme, and ALAD gene has many single nucleotide polymorphisms. A certain genetic polymorphism of ALAD gene has been suggested to modify the kinetics and distribution of lead [1,6,7,8]. Wetmur *et al*. [9] showed that this polymorphism results from a G-to-C transversion at position 177 in the coding region of the ALAD gene, leading to two alleles (ALAD1 and ALAD2) and three phenotypes, designated ALAD 1-1, ALAD 1-2 and ALAD 2-2. The ALAD2 allele contains a substitution of asparagine for lysine. These two alleles determine three isozymes, all of which display similar activities but have different charges. Asparagine is a neutral amino acid, whereas lysine is positively charged. Therefore, ALAD 1-2 heterozygotes produce an enzyme that is more electronegative than that of ALAD 1-1 homozygotes, and ALAD 2-2 homozygotes produce an enzyme that is more electronegative than that of ALAD 1-2 heterozygotes. This forms the basis of the electrophoretic technique originally used to identify the polymorphism and phenotype individuals [4]. Several studies have suggested that carriers of the ALAD2 allele, which is less common than the ALAD1 allele, would have higher blood lead concentrations than non-carriers, thereby increasing their susceptibility to lead toxicity [7,8]. Nevertheless, Smith *et al.* [10] suggested that the impact of the ALAD G177C polymorphism on lead only occurred at high levels of exposures, becoming insignificant at lower levels.

Other characteristics, such as race, years of occupational exposure, years of lead exposure and serum ion levels may also serve as factors of influence or confounders for lead workers in addition to genotype. However, there are few studies regarding these aspects in China. We have been following a group of lead workers (Han subjects) for several years. The goal of our work was to explore the sensitive factors that contribute to lead toxicity and to add new data of personal hygiene habits that could contribute to understanding the relationship between ALAD gene polymorphisms and lead toxicity.

## 2. Methods

### 2.1. Study Area and Participants

The study was carried out in several lead-acid battery and electric-flex manufacturing plants distributed in Southwestern China, where workers were monitored since 2002 with regular periodical medical check-ups performed by occupational health physicians. We collected data of 156 workers who were in contact with lead (51 males, 105 females, range 22–59 years, occupational exposure 1–42 years) at the time of their regular periodical medical check-ups. We used simple random sampling to avoid potential bias. All these workers were self-reported Han subjects and were observed throughout the whole study. Workers were classified into two main groups: the battery-storage workers and the cable workers. Workers with diseases such as heart disease, hypertension, diabetes, tumours, arthritis and the systolic pressure more than 21.3 kPa (160 mmHg) or the diastolic pressure more than 12.6 kPa (95 mmHg) was excluded from this study. The antecubital fossa of each worker was disinfected with an alcohol sponge 2–3 times before routine blood work. We obtained 10 mL of venous blood from each worker with disposable needles. Five mL of the venous blood sample from each worker used for determination of lead and analysis of gene polymorphism was blended with ethylene diamine tetraacetie acid (EDTA) in the anticoagulation tube. The rest of the blood samples were kept for conventional biochemical tests. Biochemical tests were carried out to detect serum calcium, iron, and zinc levels. Normal laboratory reference values for serum calcium, iron, and zinc were 2.25–2.75 mmol/L, 9.00–27.00 μmol/L and 7.65–22.95 μmol/L, respectively. All testing of blood was completed within 24 h. We obtained informed consent from all workers, and the study was approved by the West China Public Health Institute of Sichuan University.

### 2.2. Genotype Determination for the ALAD Polymorphism

Genomic DNA was extracted from blood samples by a routine phenol–chloroform method. An assay based on the polymerase chain reaction (PCR), restriction fragment length polymorphism was used to determine the genotype of the ALAD gene. Primer sequences were based on a previous study (5′-AGACAGACATTAGCTCAGTA-3′ and 5′-GGCAAAGACCACGTCCATTC-3′) [9]. PCR was done using 500 ng of genomic DNA, 0.5 μmol/L of each primer, 10× PCR buffer, 200 μmol/L of each deoxynucleotide triphosphate, 2.0 mmol/L MgCl_2_ and 1.5 units of Taq in a 25 μL reaction volume. The PCR program consisted of a 10-min denaturation step at 94 ºC, followed by 32 cycles of denaturation at 94 ºC for 30 s, annealing at 55 ºC for 30 s and synthesis at 72 ºC for 1 min. The amplified products were digested by restriction endonuclease Msp1 overnight at 37 ºC, and the fragments were separated by electrophoresis on an 8% polyacrylamide gel and visualised by silver staining. All genotypes were evaluated and agreed on by at least two persons independently. Ten percent of DNA samples were selected randomly for repeat analyses and the concordance was 100%. Subjects with the ALAD 1-1 genotype showed only a 582 bp fragment; subjects with ALAD 1-2 genotype showed 582 bp and 511 bp fragments; subjects with the ALAD 2-2 genotype showed only a 511 bp fragment. 

### 2.3. Determination of Lead in Blood and Urine

Venous blood was taken from study subjects and drawn into heparin-containing tubes. Urine samples were taken in the morning of the exam day. The blood and urine lead levels were measured by graphite furnace atomic absorption spectrometry (GFAAS). Normal laboratory reference values for lead in blood and urine were 0.00–0.96 μmol/L and 0.00–0.34 μmol/L, respectively. 

### 2.4. Questionnaire and Clinical Physical Examination

Short questionnaires were administered before the health examination. The questionnaires were then checked and used to collect the personal information at the time of their regular periodical medical check-ups. The investigation content included: (1) the general situation of lead exposure workers, such as gender, nationality, age, tenure and the lead occupational exposure, length of education, smoking history, medical history, drinking history, the family history and so on; (2) the self-conscious symptom survey, which assessed the performance of the nervous and digestive systems. The physical examination project mainly included general medical checks and specialised physical examinations for lead toxicity. We used the body mass index (BMI) to calculate the body fat level. The computation formula is as follows: BMI (kg/m^2^) = weight/height^2^. 

### 2.5. Statistical Analysis

Descriptive analysis: To summarise data, descriptive statistics were used to calculate the means of continuous variables such as blood-lead levels, age, working duration and biochemistry data. We also determined the dispersion of these data and whether the skewed variables were transformed into normal distributions. For category variables such as gender, smoking and alcohol consumption, job title, rates and proportions were used.

Comparative analysis: Subjects were divided into groups depending on the genotypes and jobs. Analysis of variance was used to examine the difference in the blood and urine lead level, as well as other confounders among the groups. The chi-square test was used to show the difference between nominal variables among the groups.

The data were analysed using the commercially available SPSS software (SPSS for Windows, version 11.0, SPSS Inc. Chicago, IL, USA). Results are expressed as means±standard deviation (SD). *P* values < 0.05 were considered to be statistically significant. 

## 3. Results

Altogether, we collected data from 156 workers who were in contact with lead at the time of their regular periodical medical check-ups. Research found the ALAD 1-1 genotype in 146 people (93.59%) and the ALAD 1-2 genotype in 10 people (6.41%); the ALAD 2-2 genotype was not detected. ALAD1 and ALAD2 allele frequency were 0.9679 and 0.0321, respectively. The distribution of genotypes for the ALAD polymorphism showed no deviation from the Hardy-Weinberg equilibrium (*P* > 0.05). The ALAD 1-1 genotype was found in 50 males and in 96 females; the ALAD 1-2 genotype was found in one male and nine females. According to Fisher’s exact probability method, there was no notable disparity in the distribution of G177C ALAD genotypes with respect to gender (*P* > 0.05).

The lead-exposed workers in this study were 35.2 years old on average with average occupational exposure of 9.8 years and lead exposure of 6.9 years. There was no statistically difference (*P* ＞ 0.05) between the two ALAD genotypes with respect to workers’ age, occupational exposure and or lead exposure by the Student’s t-test. According to the workers’ age, years of occupational exposure, years of lead exposure, smoking history and drinking history, we divided these workers into several subgroups. Those subgroups included people older than 30 years or less, occupational exposure of more or less than 5 years, lead exposure of more or less than 5 years, smoking history (those smoking less than 10 cigarettes per day were excluded) more or less than 2 years daily and drinking history (those drinking less than 50 mL per day were excluded) more or less than 2 years daily. For the different ALAD genotypes, the chi-square test found no statistical significance (*P* > 0.05).

Blood and urine lead levels were determined in all workers during the laboratory examinations. Results showed that the blood and urine lead levels in workers with the ALAD 1-1 genotype were higher than those with the ALAD 1-2 genotype, and the difference of urine lead level was on the margin of statistical significance (Table 1; *P* = 0.053). In the ALAD genotype stratification, results showed that the blood and urine lead levels in battery workers were much higher than those in the cable workers, and the differences made statistically significant.

**Table 1:** Blood and urine lead levels by genotypes.

Type of Work	Lead Exposure	ALAD 1-1 Genotype			ALAD 1-2 Genotype		*P*
		n	Mean±SD (μmol/L)		n	Mean±SD (μmol/L)	
Cable Workers	Blood Lead	63	0.51±0.10		6	0.44±0.11	0.083
	Urine Lead	58	0.030±0.096		5	0.026±0.014	0.369
Battery Storage Workers *	Blood Lead	83	1.91±0.42		4	1.48±0.34	0.052
*	Urine Lead	48	0.63±0.36	*	2	0.38±0.19	0.319
Total	Blood Lead	146	1.31±0.76		10	0.86±0.58	0.040
*	Urine Lead	106	0.30±0.38	*	7^*^	0.13±0.19	0.053

^*^ Three urine samples were contaminated and detected later, but the corresponding workers were out of contact.

The analysis of the self-conscious symptom survey showed that the main symptoms of these lead-exposed workers were dreaminess (49.4%), insomnia (37.8%), dizziness (33.3%) and amnesia (28.2%). The results showed that the self-conscious symptom constituted the same sequence between the two ALAD genotypes, but the incidences of dreaminess, insomnia, dizziness and abdominal pain were much higher in those with the ALAD 1-2 genotype compared to those with the ALAD 1-1 genotype (Table 2). According to the classification of self-conscious symptom frequency, the chi-square test was performed and the results showed that the incidences of vivid dreaminess and insomnia were higher in those with the ALAD 1-2 genotype than those with the ALAD 1-1 genotype (*P* < 0.05).

**Table 2:** Self-conscious symptom incidence and rank order with different ALAD genotypes (%).

Self-conscious Symptom	Incidence	ALAD 1-1 Genotype (146)		ALAD 1-2 Genotype (10)	
		Incidence	Rank order	Incidence	Rank order
Dreaminess	49.4	46.6	1	90.0	1
Insomnia	37.8	36.3	2	60.0	2
Dizziness	33.3	32.9	3	40.0	3
Amnesia	28.2	28.8	4	20.0	5
Debilitation	23.1	23.3	5	20.0	5
Abdominal Pain	22.4	21.9	6	30.0	4
Headache	21.2	21.2	7	20.0	5
Limb Pain	20.5	20.5	8	20.0	5
Numbness	16.7	17.1	9	10.0	6
Nausea	14.7	15.1	10	10.0	6
Diarrhea	11.5	11.0	11	20.0	5
Vomit	9.0	8.9	12	10.0	6

In the ALAD genotype stratification, self-conscious symptoms were analysed between several subgroups according to the workers’ occupational exposure and length of lead exposure (Table 3). The results showed that the incidences of debilitation, amnesia and dreaminess were much higher in the ALAD 1-1 genotype subgroup with more than 5 years on the job or lead exposure (*P* < 0.05). However, the incidences of self-conscious symptoms did not show statistical significance (*P* > 0.5) between each ALAD 1-2 genotype subgroup.

**Table 3:** Self-conscious symptom incidences with different ALAD genotypes, occupational exposure and lead exposure stratification (%).

Symptom	Occupational exposure		*P*	Length of Lead Exposure		*P*
	<5 year (n)	≥5 year (n)		<5 year (n)	≥5 year (n)	
ALAD 1-1						
Insomnia	30.9 (17)	39.6 (36)	0.292	26.5 (22)	49.2 (31)	0.005
Debilitation	12.7 (7)	29.7 (27)	0.019	14.5 (12)	34.9 (22)	0.040
Amnesia	16.4 (9)	36.3 (33)	0.010	18.1 (15)	42.9 (27)	0.001
Dreaminess	34.5 (19)	53.8 (49)	0.023	38.6 (32)	57.1 (36)	0.026
ALAD 1-2						
Insomnia	66.7 (2)	57.1 (4)	>0.5	57.1 (4)	66.7 (2)	>0.5
Debilitation	33.3 (1)	14.3 (1)	>0.5	14.3 (1)	33.3 (1)	>0.5
Amnesia	0.0 (0)	28.6 (2)	>0.5	14.3 (1)	33.3 (1)	>0.5
Dreaminess	100.0 (3)	85.7 (6)	>0.5	85.7 (6)	100.0 (3)	>0.5

During the physical examination of these lead-exposed workers, weakness of the tendinous reflex was found in two cases (1.3%), and arrhythmia was found in one case (0.6%). Regarding stratification with respect to the type of work, the pressure, pulse and BMI of workers were compared between the two different genotypes, but Student’s t-test found no statistical significance (*P* > 0.05).

Conventional biochemical tests were carried out to study the relationship of serum calcium, iron, and zinc levels to ALAD genotype. Results showed that serum iron and zinc serum levels in workers with the ALAD 1-2 genotype were higher than those with ALAD 1-1 genotype. With regard to the type of work stratification, the analysis results showed that the serum calcium level in cable workers with the ALAD 1-1 genotype was lower than those with the ALAD 1-2 genotype, and the serum zinc level was higher in those with the ALAD 1-1 genotype. The serum iron and zinc levels in battery storage workers with the ALAD 1-2 genotype were higher than those with the ALAD 1-1 genotype. All these differences made statistically significant (Table 4; *P* < 0.05).

**Table 4:** Serum calcium, iron, and zinc levels by genotypes.

Type of Work	Serum Ion	ALAD 1-1 Genotype		ALAD 1-2 Genotype		*P*
		n	Mean±SD	n	Mean±SD	
Cable Workers	Ca	63	2.40±0.17 (mmol/L)	6	2.47±0.05 (mmol/L)	0.024
	Fe	63	31.20±2.61 (μmol/L)	6	31.48±2.51 (μmol/L)	0.802
	Zn	63	19.67±4.62 (μmol/L)	6	17.35±1.84 (μmol/L)	0.031
Battery Storage Workers	Ca	83	2.20±0.08 (mmol/L)	4	2.13±0.10 (mmol/L)	0.087
	Fe	83	21.77±9.47 (μmol/L)	4	29.73±2.28 (μmol/L)	<0.001
	Zn	83	8.60±4.45 (μmol/L)	4	14.15±0.90 (μmol/L)	<0.001
Total	Ca	146	2.29±0.16 (mmol/L)	10	2.33±0.19 (mmol/L)	0.388
	Fe	146	25.84±8.70 (μmol/L)	10	30.78±2.46 (μmol/L)	<0.001
	Zn	146	13.37±7.11 (μmol/L)	10	16.07±2.21 (μmol/L)	<0.05

In the ALAD genotype stratification, correlation analysis was performed to determine the correlation between serum calcium, iron and zinc with blood lead levels. The results indicated that the lead-exposed workers’ blood lead level negatively correlated with serum calcium, iron and zinc level, and the correlation coefficients were −0.575, −0.559 and −0.775, respectively (Table 5).

**Table 5:** Correlations between serum calcium, iron and zinc with blood lead level by genotypes.

Variance	ALAD 1-1 Genotype	ALAD 1-2 Genotype	Subgroup Combination
Ca	−0.548 ^**^	−0.763 ^*^	−0.559 ^**^
Fe	−0.572 ^**^	−0.347	−0.575 ^**^
Zn	−0.779 ^**^	−0.695 ^*^	−0.775 ^**^

^*^ *P* ＜ 0.05; ^**^ *P* ＜ 0.01.

## 4. Discussion

ALAD can catalyse the second step of heme synthesis. Accumulation of aminolevulinic acid results when ALAD is strongly inhibited by lead, which produces neurotoxic and genotoxic effects. Early studies used the phenotyping technique developed by Battistuzzi *et al.* [4] to classify individuals as having ALAD 1-1, 1-2, or 2-2. In this procedure, whole blood samples are taken and the red blood cells are isolated and lysed. Isolation and electrophoresis of the ALAD protein permits distinction between phenotypes because of the charge differences among the isozymes. In 1991, Wetmur *et al.* [9] developed the genotyping technique based on the polymerase chain used by most investigators. A 916-base-pair sequence containing the ALAD-1/2 polymorphic site is amplified and then cleaved with Msp 1. The cleavage products are then analysed by agarose gel electrophoresis. Since there was 100% genotype-phenotype correspondence, we used this technique during our investigation. Our studies also included positive and negative controls to avoid bias.

The prevalence of the ALAD-2 allele ranges from 0 to 20% depending on the population. Generally, Caucasians have the highest frequency of the ALAD-2 allele, with approximately 18% of the Caucasian population being ALAD 1-2 heterozygotes and 1% being 2-2 homozygotes [11]. In comparison, African and Asian populations have low frequencies of the ALAD-2 allele, with few or no ALAD-2 homozygotes being found in such populations [12,13]. 

Many of the studies presented that documented genotype or phenotype frequencies gave little detail about the study population (e.g., age or source of donors), making it hard to rule out any potential biases due to subject selection. Meanwhile, there is little evidence showing interethnic differences in the distribution of the ALAD gene polymorphism that result from extensive interethnic crosses between peoples from different regions, especially in heterogeneous populations such as the Chinese population. 

The sole population-based study in Chinese was conducted by Hsieh *et al.* [12] in a Taiwanese population (n = 660). They measured blood and found the ALAD 1-1 genotype in 95.5%, the ALAD 1-2 genotype in 4.4%, and the ALAD 2-2 genotype was detected in 0.2%. ALAD1 and ALAD2 allele frequency were 0.976 and 0.024, respectively. In our study, we ensured that all participants were Han subjects, and the distribution of ALAD variants were consistent with Hsieh’s reported from Taiwan.

Early studies conducted on the ALAD polymorphism and lead poisoning focused on differences in blood lead levels by genotype in populations with different occupations. Ziemsen *et al.* [14] were the first to describe differences in blood lead levels by genotype. They found that lead-exposed workers with the ALAD 1-2 genotype had higher blood lead levels than ALAD 1-1 homozygotes and that ALAD 2-2 homozygotes had highest blood lead levels. Wetmur *et al.* [15] found similar results.

Schwartz *et al.* [16] found that the ALAD-2 allele was not clearly associated with higher blood levels. Smith *et al.* [10] found no association between the ALAD genotype and blood lead levels, which implied that the ALAD genotype may be a modifier of blood lead level only at high blood lead concentrations. However, Shaik *et al.* [17] reported blood lead levels did not differ significantly among ALAD 1-1, 1-2, and 2-2 genotypes.

In our investigation, results showed that workers’ with the ALAD 1-1 genotype had blood lead level that was higher than those with the ALAD 1-2 genotype. The findings should be discussed with caution due to the very low number of ALAD 1-2 genotypes present in this population. And most ALAD 1-2 individuals (nine out of ten) are females. Thus the lower blood lead levels of ALAD 1-2 carriers might be related to gender-specific exposure and further study was needed. 

The reason that elevated blood and urine lead were more common in battery storage workers was attributed to the more intensive work environment and much poorer protection against lead exposure. These issues were quite common among battery storage factories in China, especially in the southwestern part. 

Lead inhibits ALAD stoichiometrically, and ALAD inhibition results in the buildup of aminolevulinic acid. Aminolevulinic acid resembles γ-aminobutyric acid and can stimulate γ-amino-butyric acid receptors in the nervous system; this is thought to be one of the primary mechanisms of lead-induced neurotoxicity [18]. Other studies revealed that lead could interfere with catecholaminergic and particularly dopaminergic neurotransmission, inducing antioxidant defences and oxidative damage in brain [19]. Further research showed that lead may also decrease beta-adrenergic receptor density and adenylate cyclase activity directly [20,21]. 

There have limited studies mentioning about the relationship between self-awareness and ALAD genotype [22,23,24]. Self-reported symptoms may serve as effect markers of lead toxicity. Our study of the self-conscious symptom survey showed that the self-conscious symptom constituted the same sequence between two ALAD genotype, but the incidences of dreaminess, insomnia, dizziness and the abdominal pain were much higher in those with the ALAD 1-2 genotype than those with the ALAD 1-1 genotype. Self-conscious symptoms were also analysed by ALAD genotype stratification. The result showed that the incidences of debilitation, amnesia and dreaminess were much higher in the ALAD 1-1 genotype subgroup especially with tenure or lead exposure greater than 5 years. Schwartz *et al.* [16] reported similar findings. We proposed that the ALAD-2 subunit of the protein could maintain lead in a nonbioavailable form, such that these individuals were protected from lead’s effects and could tolerate longer exposures to lead than those with ALAD-1 subunit.

Interactions between calcium and lead were previously documented [25,26]. Competition for Ca-binding proteins may underlie a mechanism for lead absorption. Pires *et al.* [27] reported that calcium supplementation during lactation appeared to blunt the lactation-induced increase in maternal blood lead. Ettinger *et al*. [28] reported similar findings. Varnai *et al.* [29] also concluded that higher calcium intake might be a way of efficient reduction of lead absorption during the suckling period. However, Markowitz *et al.* [30] found that calcium supplementation had no significant effect on the change in blood lead levels.

There is evidence that iron could be a neuroprotective factor in lead-induced attention deficit disorder with hyperactivity [31]. Muwakkit *et al.* [32] found that iron deficiency was associated with elevated blood lead levels. Wang *et al.* [33] reported that iron supplement among lead-exposed rats maintained the normal ultra-structure of the blood-brain barrier and restored the expression of occluding to normal levels. Kim *et al.* [34] confirmed the effectiveness of dietary iron intake as a secondary preventive intervention against lead toxicity. However, Kordas *et al.* [35] found that iron and zinc supplementation did not improve behaviour in children exposed to lead. Serwint *et al.* [36] reported there was no difference in iron status between children with low or moderate lead exposure.

Zinc is an important element that can prevent lead poisoning [37]. The protective effect may be due to competition between lead and zinc or displacement of lead by zinc. Batra *et al.* [38] found that zinc supplementation ameliorated lead-induced testicular damage both at the cellular and subcellular level. However, Rico *et al.* [39] revealed that daily supplementation with iron and/or zinc might be less effective in improving cognition in school children than in younger children. 

We evaluated the interaction of serum calcium, iron, and zinc levels with blood lead levels in our study. In the ALAD genotype stratification, correlation analysis indicated that the lead-exposed workers’ blood lead levels negatively correlated with serum calcium, iron and zinc levels, and was consistent with earlier studies [40]. Furthermore, the interactions between zinc and lead would be significant when blood lead levels are relatively high. 

## 5. Conclusions

In conclusion, the distribution of G177C ALAD variants in Han subjects of the Chinese population is consistent with Hsieh’s reported from Taiwan [12]. Workers with the ALAD 1-1 genotype were associated with higher blood lead level than those with the ALAD 1-2 genotype. The self-conscious symptom survey showed that the incidences of debilitation, amnesia and dreaminess were much higher in those had more than five years of tenure or contact with lead on the job within the ALAD 1-1 genotype subgroup. The lead-exposed workers’ blood lead level negatively correlated with serum calcium, iron and zinc levels. Therefore, we recommend that an adequate intake of dietary calcium, iron, and zinc or the calcium, iron, and zinc supplementation should be routinely prescribed to prevent toxic effects of lead exposure in Chinese lead workers. Moreover, the work environment and protection against lead exposure should be improved and ensured, especially in Southwestern China. 
